# A Philosophical Review of School Nursing Framed by the Holistic Nursing Theory of Barbara Dossey

**DOI:** 10.1177/08980101211006615

**Published:** 2021-04-15

**Authors:** Pernilla Garmy, Eva K. Clausson, Ann-Christin Janlöv, Eva-Lena Einberg

**Affiliations:** 4342 Kristianstad University Clinical Health Promotion Center, Lund University; Kristianstad University

**Keywords:** holistic nursing theory, school nursing, Barbara Dossey

## Abstract

This article is a philosophical review of school nursing and its constructs framed by Barbara Dossey’s holistic nursing theory. The author describes the application of holistic nursing theory within the school nurse’s area of activity. The review suggests that holistic nursing theory can be applied in several areas of school nursing. School nurses have a multifaceted occupation that includes meetings with students, parents, and school staff. Barbara Dossey’s holistic nursing theory offers the school nurse tools to deal with people’s varied experiences, feelings, and needs.

## Introduction

Holistic nursing is derived from the philosophies of holism and humanism (Frisch & Rabinowitsch, 2019). Dossey (2016) has developed a holistic nursing theory (see Table 1). Central to Dossey’s holistic nursing theory is Florence Nightingale’s thoughts on nursing (Nightingale, 1859, 1992) and the philosopher Ken Wilbur’s holistic view of reality (Wilber, 1999). Dossey (2016) reported that holistic nursing theory does not exclude other nursing theories. These can be advantageously included in this “grand theory.” The concept of “person” is important in holistic nursing theory, and the theory has several points of contact with person-centered nursing (McCormack & McCance, 2006; McCormack et al., 2017).

**Table 1.** The Holistic Nursing Theory.

The holistic nursing theory consists of five components as well as aspects/elements of these five
1. Healing
2. Metaparadigm of nursing theory a. Nurse b. Person(s) c. Health d. Environment (society)
3. Patterns of knowing a. Personal b Empirical c. Socio-political d. Ethics e. Aesthetics f. Not-knowing
4. The four quadrants a. Internal individual factors—feelings and experiences b. External individual factors—behavior and physical symptoms c. Internal collective factors—group culture and values d. External collective factors—organization and system
5. Conclusion of all components

In person-centered nursing, the care seeker is seen as an equal partner involved in the planning of their care (Morgan & Yoder, 2012). The nurse meets a unique person who, in addition to needing care, is also capable and has resources. Both Dossey’s nursing theory and person-centered nursing show the importance of the nurse as having both professional and social competence. The nurse is open and committed to the personal meeting, has self-awareness, and is aware of her/his own values. The person’s story is central, and the social context is important because the person is dependent on this context, for example, who they interact with and their surroundings.

Furthermore, the importance of the societal perspective is emphasized with the attitudes, norms, laws, and guidelines that affect care. Nurturing holistically is the basis for both Dossey’s nursing theory and person-centered nursing. Holistic nursing is founded on the values of integrality and awareness of whole-people and whole-system interconnectedness. This is closely linked with the broader global health agendas and initiatives of our time such as the United Nations Sustainable Development Goals, which seek to improve human, animal, and planetary health (Rosa et al., 2019).

The school nurse’s work includes complex interactions with students, guardians, and school staff. The school is an environment where teachers focus on learning while the school nurse promotes health and prevents disease. Morberg et al. (2012) investigated the school nurse’s role based on Bourdieu’s theories about habitat and found that it can be a challenge for the school nurse to find her/his way in this environment. In addition, students’ needs are often difficult to meet, and conditions for good work can vary depending on the context. There is a need for school nurses to express their values and to make their philosophy clear (Garmy, 2020).

Rosa et al. (2020) claimed that nursing theory informs knowledge development and theory-guided practice. The holistic nursing theory described by Dossey (2016, 2008) has been applied in very different areas such as end of life care and rehabilitation (Dossey & Keegan, 2016). However, to the best of our knowledge, there is no scientific review of its value in school nursing. This article is a philosophical review of school nursing and its constructs, framed by Barbara Dossey’s holistic nursing theory.

Dossey does not mention school nurses in her texts. She consistently writes “nursing” and “nurse.” When we refer to Dossey’s own texts, our intention is to use the word nurse and nursing, but we write as school nurses when we apply Dossey’s theory in school health care.

### Healing—the First Component in the Holistic Nursing Theory

The core of the holistic nursing theory is healing, which includes knowledge, ability, and attitude. Healing occurs on a physical, mental, social, and spiritual/existential level, and it is important that these parts interact to create balance. Developing knowledge, ability, and attitudes about healing is a lifelong process to reach a deeper knowledge where we meet our fears and also learn confidence in life, creativity, passion, and love. All living creatures have innate healing abilities. Healing work is not synonymous with cure. It is not always possible to cure, but one can promote healing throughout life. Intentions are a key factor in the process. Here, intention means a conscious intention to do a specific thing or to act in a specific way, that is, to be involved in, plan, or try to perform an action.

The concept of health can be derived from wholeness and healing (Medin & Alexanderson, 2000). The school nurse’s work basically consists of healing and health-promotion work including meetings with students and parents/guardians focused on health dialogues, vaccinations, teaching, and spontaneous visits. Examples of a healing meeting can be in a health dialogue when the school nurse takes the time to listen actively, and, together with the child or adolescent, finds health-promoting strategies. Tinnfält (2019) describes the health dialogue from a student’s perspective especially with a focus on mental illness. Students want to meet a school nurse who believes in them, shows them respect, and is honest but also personal. They want a dialogue between two equal people.

### Metaparadigm of Nursing Theory—the Second Component in the Holistic Nursing Theory

In the second component of the holistic nursing theory, the consensus concepts within nursing—or the nursing metaparadigm—are used; namely, nurse/nursing, person, health, and environment. The concepts are coherent and interdependent. A change in one domain will lead to change in the others as well.

The nurse is a part of the health-promoting process and contributes herself/himself in relation to the person or group of people that he/she meets. This reinforces the meaning and experience of unity and belonging. The recognition of the consensus concepts in nursing has consequences for the school nurse’s work. First, the school nurse needs to see her/his own self in relation to the children and adolescents he/she meets. Studies have shown that school nurses find it meaningful but also challenging to meet students in difficult situations. Such work requires further training and supervision (Bonde et al., 2014; Jonsson et al., 2017; Berg & Clausson, 2019; Musliu et al., 2019; Berglund Melendez et al., 2020).

A person is defined as an individual (patient or relative) to whom the nurse must respond with respect for the person’s subjective experience of health, values, sexual orientation, and personal preferences. The goal of holistic nursing is a person who feels whole and is in balance, that is, an integrated person. The definition also includes a nurse who interacts with a colleague or other health care professional. In school health care, the person is a student or parent/guardian.

The concept of a person makes a person-centered approach visible in relation to the students and their parents/guardians. A person-centered approach can help treat people with respect to their subjective experiences. The health dialogue can be a powerful tool to provide support to children and adolescents, but it is crucial that the student’s own experience of their situation be at the center (Golsäter et al., 2011; Wigert et al., 2019).

Health consists of physical, mental, emotional, social, and existential dimensions. These dimensions are different facets of a holistic view of health that includes seeing illness and death as a natural process in life. The goal of nursing is to promote different dimensions of health for integrated health. Considering health from its various dimensions of physical, mental, emotional, social, and existential natures becomes important in the encounter with children and adolescents. The school nurse’s task is to promote the health of the students; clearly, health and learning are related to each other. Good health improves the opportunities to achieve the learning objectives and to leave school with suitable grades that increase the conditions for future good health (Einberg & Wilhsson, 2019a, b). Children and adolescents report that health is promoted through community and good relationships with family and friends. Security and trust are important as well as opportunities for activities that engage and/or provide recovery (Einberg & Wilhsson, 2019a, b).

The school nurse often sees children and adolescents with different types of pain such as headaches or stomach aches (Høie et al., 2017; Wigert et al., 2019). Physical pain is more common in children and adolescents who are also depressed (Borgman et al., 2020) or bullied (Garmy et al., 2019a). The use of painkillers is also more common in children and adolescents who are depressed (Hena et al., 2019) or bullied (Garmy et al., 2019b). A recent review by Ragnarsson et al. (2020) showed that students with pain have more difficulty achieving school-learning goals and obtaining suitable grades; other mediating factors such as school absenteeism and concentration problems are also important. The school nurse’s assignment includes supporting students so that they can achieve their learning objectives. In the individual health dialogue, the school nurse can detect ill-health early on but also support the students in actions that promote health (Einberg & Wilhsson, 2019a, b). The Calgary models of family nursing (Shajan & Snell, 2019) can be helpful in health-supporting family dialogues (Clausson, 2019) where the family is involved and invited to dialogues. Models of family nursing can reduce suffering in both students and family members.

The environment consists of both internal and external aspects. The inner environment includes the person’s emotions as well as their mental, emotional, and existential dimensions. The external environment includes things that can be observed and measured and that are related to what is physical and social in society. An integrated environment means that the internal and external aspects are in harmony with each other.

The environment in the school nurse’s work is the school where they perform their activities. It is both the school nurse’s office and the school environment where the students stay. However, it is also the context in which the students stay outside of school, that is, the home environment and the surrounding society. A study where school nurses assessed students’ physical and mental health showed that students in schools of vulnerable areas have worse physical and mental health than other areas in Sweden (Ellertsson et al., 2017). In another study, school nurses say that the school nurse’s office and the waiting room can be like an oasis at school for students with mental illness (Jonsson et al., 2017). A school environment where students experience participation, support, and feel safe and comfortable has shown a connection to better self-reported student health (Vaičiūnas & Šmigelskas, 2019; Warne et al., 2017).

### Patterns of Knowing—the Third Component in the Holistic Nursing Theory

The third component of the holistic nursing theory covers different areas of knowledge in nursing. These six areas of knowledge are the personal, the empirical, the aesthetic, the ethical, the not-knowing, and the socio-political. These areas of knowledge help nurses to be present in the present, to integrate art and science, and to develop ethical sensitivity of thought and action.

Personal knowledge is linked with the first principle in component 5 and can be developed through art, meditation, dance, music, stories, and other means of expression in daily and professional life. This is about the nurse’s responsibility for her/his personal development process. For example, the importance of self-knowledge in school nursing was highlighted in a study of school nurses’ experience of meeting unaccompanied refugee children (Musliu et al., 2019), students with mental illness (Jonsson et al., 2017), and those with neurodevelopment disorders (Berglund Melendez et al., 2020). The results indicate a need for professional guidance. Today, however, there is a lack of scientific studies on professional guidance in the school nurse’s activities (Berg & Clausson, 2019).

Empirical knowledge is nursing science that focuses on scientific competence in education and clinical practice. It is expressed in models and theories and can be integrated into evidence-based practice. Empiricism is experienced through observation, measurement, and verification. A need for knowledge about theories and models related to the health-promoting work performed by school nurses has emerged (Reuterswärd & Lagerström, 2010).

Several studies have shown the importance of the school nurse’s professional experience, for example, in meetings with students with mental illness (Jonsson et al., 2017) and with students who have a parent with a serious illness (Golsäter et al., 2017). Studies of school nurses’ experiences can be translated into theories and models. Borup (2002) developed a model for health dialogues based on interviews with school nurses. Clausson (2019) tested existing models for family-focused nursing within the school nurse’s area of activity to promote school children’s mental health. Bonde et al. (2014) studied how school nurses have adapted and integrated motivational interviewing (MI) into their practice. Theories and models can also be used to understand school nursing practices, for example, by using the health belief model to understand school nurses’ asthma management (Quaranta & Spencer, 2015).

Aesthetic knowledge is nursing as an art and focuses on exploring experiences and meaning in life and includes authentic presence where the nurse is a facilitator of the healing processes. This is a combination of knowledge, experience, and intuition. With the help of aesthetic knowledge, the nurse and the person under care can explore experiences of life, health, illness, and death. In the model of health dialogues by Borup (2002), authenticity is one of the four central factors (in addition to competence, reflective openness, and supportive environments); this means that the school nurse shows an honest and genuine interest in the student’s story. In focus group interviews, children expressed that enjoying what is beautiful and good can promote their health (Einberg, 2016). Furthermore, dance can reduce symptoms such as headaches, dizziness, stress, and anxiety (Duberg et al., 2020). By sharing aesthetic experiences such as beauty in nature and listening to the student’s stories about what is experienced as meaningful, the school nurse can, in dialogues with students, support the student in lifestyle choices that promote health.

Ethical knowledge is translated into moral action. It is nursing knowledge that focuses on behaviors, expressions, and ethical and moral dimensions. This emphasizes respect for the person, the family, and society that encourages relationships including attention, communication, and moral action. An ethical dilemma could occur when the school nurse and the parents do not see the same problem. Bonde et al. (2014) gave an example of overweight/obesity if the family does not find it to be a health issue. The school nurse is then between retaining the spirit of MI, that is, respecting the family’s autonomy, and her/his professional role and responsibility for the health of the child (Bonde et al., 2014).

Ethical dilemmas in dialogues can arise when students reveal misconduct in the home and concurrently express that it must not be passed on (Tinnfält, 2019). Here, different ethical principles can come into conflict with each other and with legislation. The obligation to report to social services violates confidentiality. It is important that the student be involved and can understand what must happen, but it can be difficult to draw the line as to when there is a duty to report. Examples of ethically difficult situations can be documentation of sensitive information in the students’ journal, for example, relationship problems in the family. School nurses have expressed difficulty in selecting and formulating what should be documented especially because parents/guardians can request access to the documentation (Clausson, 2020; Clausson et al., 2015).

Not-knowing is the ability to be present in the moment with a health-promoting purpose without having preconceived answers. It includes authenticity, mindfulness, openness, surprise, and the discovery of oneself and others in the subjective and intersubjective realm. Not-knowing allows new solutions, opportunities, and insights to emerge. Once again, Borup’s model for health dialogues can be mentioned because the model describes how a communicative space for learning about health can be created. The health dialogue invites an open reflection where the student and school nurse think aloud together so that the student herself/himself will find solutions to promote her/his health (Borup, 2002). Golsäter et al. (2012) argued that the health dialogue should be student-centered and based on the student’s situation and needs, which can be compared with person-centering (McCormack & McCance, 2006; McCormack et al., 2017) where the person’s story is central, and openness and commitment are important.

Socio-political knowledge is about the importance of context in the form of social, economic, geographical, cultural, political, historical, and other key factors in theoretical, evidence-based practice, and research. The school nurse needs to be aware of, and have knowledge of, the local conditions that affect the students’ health. Einberg and Wihlsson (2019a, b) applied ecological systems theory to the school nurse’s field of work and described how the work is managed and affected by laws, norms, and values at the societal level. The work is also affected by resources at the organizational level as well as relationships and environments in the family and school. Knowledge of current health-related living habits, living conditions of the students, as well as the resources available in the local area provide a basis for the school nurse’s local health-promotion work. From previously being an assistant to the school doctor, the school nurse’s profession has become professionalized and independent of its own area of knowledge that rests on a scientific basis (Morberg et al., 2012).

### The Quadrants—the Fourth Component in the Holistic Nursing Theory

**Figure 1.**
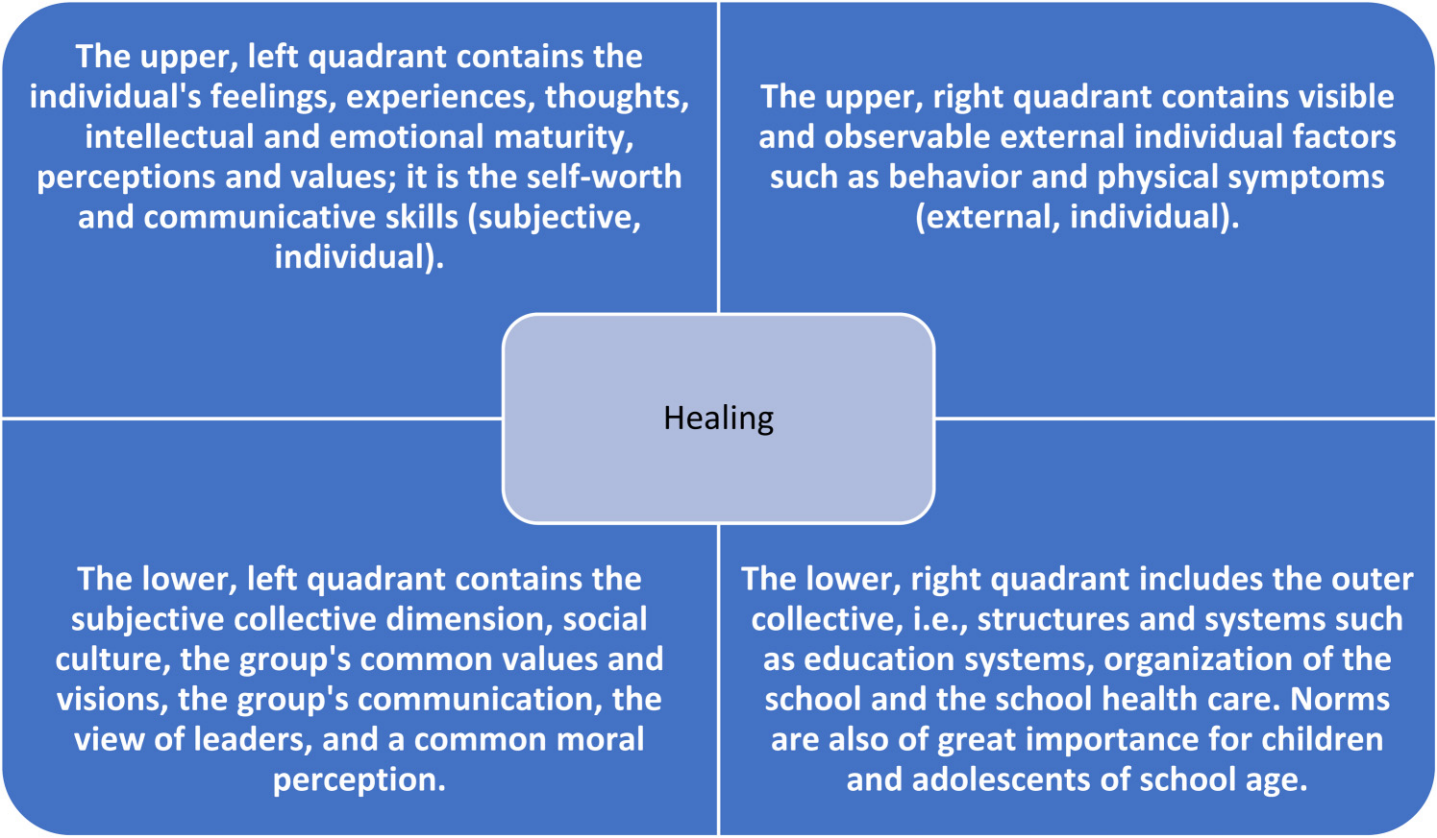
In the fourth component, Dossey adapted a model by Wilber (1999) where reality is described from four perspectives that relate to each other. These perspectives are made visible in four quadrants and include both internal and external aspects as well as individual and collective aspects.

In the fourth component, Dossey adapted a model by Wilber (1999) where reality is framed by four perspectives that relate to each other. These perspectives are described and made visible in four quadrants offering a holistic picture of a person and/or situation. They include both internal and external aspects as well as individual and collective aspects (Figure 1). Wilber’s model has been used in health care research to gain a holistic view of identifiable circumstances needing improved interventions (Janlöv et al., 2016). Høie et al. (2017) proposed one example of how school nurses view the students through the four quadrants while also challenging the work in line with this understanding. The school nurses in this study reported that the pain among adolescents was social, physical, and psychological phenomena, and they had an ambivalent attitude to medicalizing pain. Høie et al. (2017) concluded that school nurses maintained the practice of medical examinations despite a biopsychosocial understanding of pain.

### All Quadrants, all Levels—the Fifth Component in the Holistic Nursing Theory

In the fifth component, all quadrants and all levels are combined and start from the healing core in the middle while placing the four quadrants around this center. An understanding of the complexity of this component is deepened through personal and professional maturity and competence development. One can use holistic nursing theory, concepts, and practices that are related to the body, self, and spirit integrated into the self, culture, and nature. The quadrants are raised to a new level and are called the four integrated nursing principles. Here, the focus is on the caregiver (i.e., the school nurse). That deepened self-development in relation to each quadrant is required for professional healing capacities. In other words, you must start with your own health to become trustworthy as a health promoter.

The first principle includes the “self,” that is, the subjective space. Each of us must explore our own health, well-being, and thoughts about future death through personal development. This describes how the nurse handles stress and suffering; it is about self-care such as conscious presence and reflection. To be strengthened in the role of a professional school nurse, it is necessary to develop and renew oneself (Berg & Clausson, 2019). Participating in regular systematic clinical reflection provides such an opportunity. Increased self-efficacy among school nurses is associated with increased involvement in comprehensive childhood obesity prevention (Quelly, 2014).

The second principle includes “we” and concerns relations and the intersubjective area. It can be about meeting the suffering of people we meet on a deeper level and then being able to share these difficult life processes. This requires the ability to be actively present and conveying empathy. This was demonstrated by school nurses working with students with mental health problems (Jonsson et al., 2017) and unaccompanied refugee children (Musliu et al., 2019). School nurses need to build a solid relationship with students for them to disclose bullying (Pigozi & Jones Bartoli, 2016) especially since the school nurses did not feel confident in identifying subtle and/or cyber bullying. Pigozi and Jones Bartoli (2016) reported the lack of training and the lack of time for preventive work as problematic.

Sensitive issues such as sexual abuse affect school nurses emotionally with feelings of frustration, despair, and anger (Kraft & Eriksson, 2015). They are also professionally vulnerable with feelings of ambivalence. Working alone with professional secrecy prevented them from seeking support in their work. A lack of time also resulted in a lack of power (Engh Kraft et al., 2017).

A situation that the school nurses brought up as stressful was having to handle being caught in the middle between the relationship and obligations toward the student versus teacher or parent—this situation could become even more complex via language difficulties and/or cultural diversity (Hansson et al., 2012).

The third principle “it” is about the individual and objective area. It is about professionalism, behavior, and actions that lead to healthy lifestyles and a healthier body. It is also about teaching, evaluation, nursing skills, and interventions. The school nurses in the study by Hansson et al. (2012) reported that students found it easier to approach the nurse for help in sensitive matters such as eating disorders, substance use, contraception, or sexually transmitted diseases instead of other professionals.

School nurses’ work includes a commitment on a population level. The Public Health Intervention Wheel (PHIW) is a framework for school nursing practice in a study by Schaffer et al. (2016). The PHIW includes five colored wedges of intervention grouped with a similar focus including healthy lifestyles, teaching, and evaluation. The PHIW is evidence-based and may be helpful for school nurses in their health-promoting work.

The fourth principle “its” includes collective external factors such as the health care organization and the political system. This involves working in interprofessional teams to offer effective health care and coordinating care to create health-promoting care environments and workplaces. School health care is based on work in interprofessional teams. School nurses’ experience of shared responsibility in school-based interprofessional teams was studied by Reuterswärd and Hylander (2017). The school nurses reported a lack of clarity of their role in the interprofessional teams, which needs to be clarified because there are several opportunities to collaborate and satisfy the needs of school children.

For example, migration is a global concern (Clausson & Cowell, 2019), and collaborations to develop interventions addressing the needs of migrant schoolchildren are of high priority. This can be done together with school nurses in their daily work to promote the health of school children (Clausson & Arvidsson, 2020). Several studies highlight the benefits and challenges of collaborative work with interdisciplinary staff, for example, in work with students having intellectual and developmental disabilities (Singer, 2013; Berglund Melendez et al., 2020), type 1 diabetes (Wang & Volker, 2013; Thorstensson et al., 2016), children with unexplained physical symptoms (Shannon et al., 2010), and with staff and adequate others to prevent bullying among students (Pigozi & Jones Bartoli, 2016).

### Strengths and Limitations

It is a challenge to summarize and concretize a major theory. Our intention is not to convey Dossey’s holistic nursing theory in all its complexity. Those who want deeper knowledge should go to Dossey’s original works. Nevertheless, there have been requests from clinically active school nurses for help in adopting Dossey’s holistic nursing theory, and this article helps to meet this need.

## Conclusions

School nurses have a multifaceted job that includes meetings with students, parents, and school staff. By working on the basis of Barbara Dossey’s holistic nursing theory, the school nurse is given tools to deal with phenomena such as people’s experiences, feelings, and needs.
